# Nonmaximal entanglement of photons from positron-electron annihilation demonstrated using a plastic PET scanner

**DOI:** 10.1126/sciadv.ads3046

**Published:** 2025-04-30

**Authors:** Paweł Moskal, Deepak Kumar, Sushil Sharma, Ermias Yitayew Beyene, Neha Chug, Aurélien Coussat, Catalina Curceanu, Eryk Czerwiński, Manish Das, Kamil Dulski, Marek Gorgol, Bożena Jasińska, Krzysztof Kacprzak, Tevfik Kaplanoglu, Łukasz Kapłon, Tomasz Kozik, Edward Lisowski, Filip Lisowski, Wiktor Mryka, Szymon Niedźwiecki, Szymon Parzych, Elena P. del Rio, Martin Rädler, Magdalena Skurzok, Ewa Ł. Stepień, Pooja Tanty, Keyvan Tayefi Ardebili, Kavya Valsan Eliyan

**Affiliations:** ^1^ Faculty of Physics, Astronomy and Applied Computer Science, Jagiellonian University, S. Łojasiewicza 11, 30-348 Kraków, Poland.; ^2^ Total-Body Jagiellonian-PET Laboratory, Jagiellonian University, 30-348 Kraków, Poland.; ^3^ Center for Theranostics, Jagiellonian University, 31-034 Kraków, Poland.; ^4^ INFN, Laboratori Nazionali di Frascati CP 13, Via E. Fermi 54, 00044, Frascati, Italy.; ^5^ Institute of Physics, Maria Curie-Sklodowska University, Pl. M. Curie-Sklodowskiej 1, 20-031 Lublin, Poland.; ^6^ Cracow University of Technology, Faculty of Mechanical Engineering, 31-864 Kraków, Poland.

## Abstract

In state-of-the-art positron emission tomography (PET), information about annihilation photon polarization is unavailable. Here, we present a PET scanner built from plastic scintillators, where annihilation photons primarily interact via the Compton effect, providing information about both photon polarization and propagation direction. Using this plastic-based PET, we determined the distribution of the relative angle between polarization planes of photons from positron-electron annihilation in a porous polymer. The amplitude of the observed distribution is smaller than predicted for maximally quantum entangled two-photon states but larger than expected for separable photons. This result can be well explained by assuming that photons from pick-off annihilation are not entangled, while photons from direct and parapositronium annihilations are maximally entangled. Our result indicates that the degree of entanglement depends on the annihilation mechanism in matter, opening avenues for exploring polarization correlations in PET as a diagnostic indicator.

## Teaser

Nonmaximal entanglement of annihilation photons is observed in a porous medium with a polarization-sensitive J-PET scanner.

## INTRODUCTION

Positron emission tomography (PET) is an established imaging technique for noninvasive in vivo diagnosis of disease in clinical practice (*1*–*3*). Its potential for quantitative assessment of metabolic alteration in biological tissues makes it useful for various medical applications by assessing physiology (functionality) of human organs or tissues (*4*). In PET, a biomolecular tracer labeled with a positron (*e*^+^) emitting radionuclide is administered into the human body. The emitted *e*^+^ interacts with electron (*e*^−^) in the tissue and annihilates predominantly into two 511-keV photons moving in opposite directions.

**Fig. 1.**
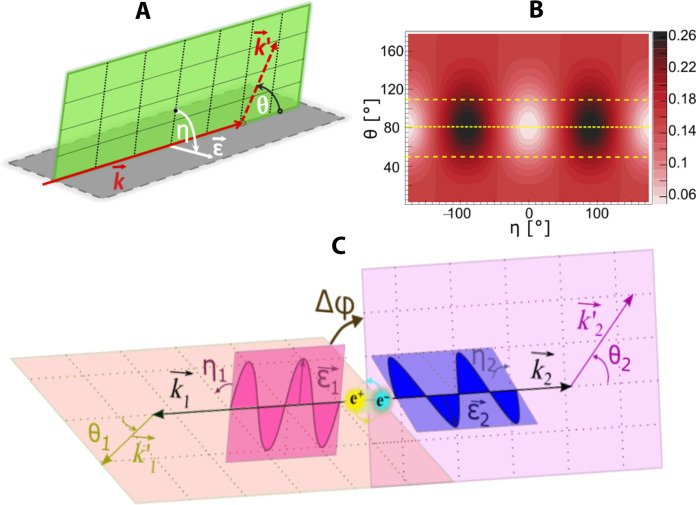
Relation between scattering angle and photon polarization in the Compton scattering of annihilation photons. (**A**) Pictorial illustration of the photon-electron Compton scattering with the definition of the photon scattering angle (θ), photon polarization ($\vec{\varepsilon }$), photon momentum before ($\vec{k}$) and after ($\vec{k\prime }$) the scattering, and the angle (η) between the polarization and scattering planes. (**B**) Normalized Klein-Nishina cross section for 511-keV photons as a function of angles η (horizontal axis) and θ (vertical axis). For normalization, the two-dimensional plot (η and θ) was weighted such that for each value of θ, the integral of the cross section over the entire range of η from −π to π is equal to unity. The yellow dotted line indicates the value of θ = 82°, for which visibility is maximum (*15*), and dashed lines indicate a range of high visibility for θ = 82° ± 30° (*23*). (**C**) Schematic representation of annihilation photons and their Compton scattering, including polarization and scattering planes. θ_1_ and θ_2_ denote the Compton scattering angles. η_1_ and η_2_ denote angles between the scattering and polarization planes. Δφ represents the angle between the scattering planes of annihilation photons and thus is a measure of the relative angle between their polarization planes.

The principle of PET is based on the registration of places and times of interactions of these two photons, and reconstruction of the site of annihilation along their direction of propagation referred to as the line of response (LOR). The information from the LORs is used as input to reconstruct the density distribution of annihilation points. However, annihilation photons carry more information than just about the site where they originated. In general, annihilation photons carry information in the form of energy, direction of propagation, polarization, and the degree of entanglement (*5*–*7*). Polarization of annihilation photons is not accessible by current PET systems, but in principle, it can inform us about the contributing annihilation mechanisms, which, in turn, may tell us about the cell molecular composition. In the body, a positron emitted from the isotope attached to the biomolecule may annihilate with an electron either directly or via formation of positronium (*8*, *9*). Positronium in the tissue intermediates the positron-electron annihilation in about 40% of cases (*10*–*12*). Positronium may be formed as a long-lived (142 ns) spin-one orthopositronium (oPs) or as a short-lived (125 ps) spin-zero parapositronium (pPs) (*13*). In vacuum, oPs decays into three photons (oPs → 3γ) and pPs into two photons (pPs → 2γ) (*9*). Theoretically, photons from the decay of positronium in vacuum are maximally entangled in polarization (*14*–*16*). However, in matter when positron from positronium annihilates with the electron bound to the atom, it is natural to ask the question of whether the photons resulting from this annihilation are maximally entangled (*17*–*20*). Annihilation photons have energy in the range of mega–electron volts and hence interact in matter with single electrons. Therefore, their polarization cannot be studied using optical methods. However, polarization of these high energetic photons can be estimated by Compton scattering (Fig. 1A). The Compton scattering of photons is most likely in a plane perpendicular to the polarization of the incoming photon (*21*), and therefore polarization orientation of the primary photon ($\vec{\varepsilon }$) at the moment of scattering can approximately be determined as a vector product of momentum vectors of initial $\vec{k}$ and scattered photon $\vec{k\prime }$ (*22*)

$$\vec{\varepsilon }=\frac{\vec{k}\times \vec{k\prime }}{\left|{\vec{k}}_{}\times {\vec{k\prime }}_{}\right|}$$ (1)

Figure 1B describes the distribution of η (*23*), the relative angle between the polarization plane and the scattering plane, as a function of the scattering angle θ. It indicates that, in the case of 511-keV annihilation photons, the correlation between the polarization direction $\vec{\varepsilon }$ and the scattering plane is maximal at about θ = 82°, decreasing to a negligible effect for forward (θ = 0°) and backward (θ = 180°) scattering. In general, the analyzing power (*A*_p_) of the Compton polarimeter is expressed as (*15*, *24*, *25*)

$$A_{p}={\text{sin}}^{2}\theta /\left(E/E\prime +E\prime /E-{\text{sin}}^{2}\theta \right)$$ (2)

where *E* and *E*′ denote the energy of primary and scattered photons, respectively (*25*). For 511-keV photons, the maximum value of *A*_p_ equal to 0.69 is reached at θ = 82°.

The distribution shown in Fig. 1B indicates that the Compton scattering may be effectively applied as a polarimeter in the scattering angle range of about θ = 82° ± 30°, in which the analyzing power varies from 0.69 to 0.42. This range is indicated by horizontal dashed lines. For the 2γ annihilation process (Fig. 1C), when each γ interacts via Compton scattering with an electron, one can estimate the relative angle between the polarization directions of the photons ∣η_1_ − η_2_∣ by measurement of the relative angle Δφ between the scattering planes (*23*). Bose symmetry and parity conservation in the decay of pPs imply that the state ∣ψ〉 of the resulting two photons is maximally entangled and that the photon polarizations are orthogonal to each other (*15*). In the linear polarization base, the two-photon state from pPs decay reads

$$|\psi \rangle =\frac{1}{\sqrt{2}}\left({|H\rangle }_{1}{|V\rangle }_{2}+{|V\rangle }_{1}{|H\rangle }_{2}\right)$$ (3)

where *H* and *V* correspond to the horizontal and vertical polarization directions. Taking into account the Compton scattering of each of the photons, the double Compton scattering differential cross section can be expressed as (*26*)

$$\frac{d^{2}\sigma \left(\theta_{1},\theta_{2},\Delta \varphi \right)}{d\Omega_{1}d\Omega_{2}}=\left[A\left(\theta_{1},\theta_{2}\right)-B(\theta_{1},\theta_{2})\text{cos}\left(2\left(\Delta \varphi \right)\right)\right]$$ (4)

where *A* and *B* describe the dependence of the cross section on the Compton scattering angles θ_1_ and θ_2_. At given scattering angles (θ_1_ and θ_2_), the cross section is maximal for Δφ = 90° and minimum for Δφ = 0°. The strength of the polarization correlation of the annihilation photons may be described by the *R* factor (referred to as entanglement witness), which is the ratio of the probabilities of the scattering at Δφ = 90° and Δφ = 0°. This implies

$$R=\frac{A\left(\theta_{1},\theta_{2}\right)+B\left(\theta_{1},\theta_{2}\right)}{A\left(\theta_{1},\theta_{2}\right)-B\left(\theta_{1},\theta_{2}\right)}$$ (5)

The maximum of the cross section at Δφ = 90° reflects the fact that the polarizations of the photons are perpendicular to each other. For maximally entangled photons, the value of *R* reaches a maximum of *R*_max_ = 2.84 at the scattering angles of θ_1_ = θ_2_ = 82° (*15*, *26*–*34*), while for separable photons, the maximum value of *R* is equal to *R*_sep_ = 1.63 (*32*, *33*). The shape of the distribution of the angle Δφ can, in principle, carry information about the molecular composition of annihilation sites. In matter, 2γ annihilations (used in PET) occur either by (i) direct electron-positron annihilation, (ii) via self-annihilation of pPs, or (iii) via oPs annhilation due to the interaction with surrounding electrons. The oPs, in addition to self-annihilation into three photons, may annihilate into two photons when the positron from oPs picks off an electron from the surrounding molecular environment (pick-off process) (*9*). Here, we hypothesize that the Δφ distribution may depend on the annihilation mechanism. In general, we assume that in an α fraction of 2γ annihilations, the photons are maximally entangled, and in the remaining (1 − α) fraction, the produced photons propagate independently of each other. Thus, without loss of generality, the measured Δφ distribution *F*(Δφ) may be decomposed as

$$F\left(\Delta \varphi ,\alpha \right)=\alpha F\left(\Delta \varphi ,R_{\text{max}}\right)+\left(1-\alpha \right)F\left(\Delta \varphi ,R_{\text{sep}}\right)$$ (6)

Using this formula, one can show that

$$R\left(\alpha \right)=\frac{\left[1+\alpha \left(R_{\text{max}}-1\right)/\left(R_{\text{max}}+1\right)+\left(1-\alpha \right)\left(R_{\text{sep}}-1\right)/\left(R_{\text{sep}}+1\right)\right]}{\left[1-\alpha \left(R_{\text{max}}-1\right)/\left(R_{\text{max}}+1\right)-\left(1-\alpha \right)\left(R_{\text{sep}}-1\right)/\left(R_{\text{sep}}+1\right)\right]}$$ (7)

Trying to estimate an expected effect of *R* variation in the material, we assume further that photons from the pick-off annihilations are separable (*R* = *R*_sep_), and photons from other processes (direct annihilation, pPs decay, and oPs conversion) are maximally entangled (*R* = *R*_max_). The assumption about maximal entanglement for direct annihilation is justified because previous investigations of the Δφ distribution for annihilation in metals are consistent with the predictions for maximally entangled photons (*24*), and, in metals, positrons annihilate only directly with electrons. In addition, self-annihilation of pPs theoretically leads to a pair of maximally quantum entangled photons (*14*). However, in the case when oPs annihilates via interactions with an electron from the surrounding atoms (pick-off process), the initial state will be a mixture of electron-positron states with many possible quantum numbers, and, therefore, it is plausible to assume that, in this case, for the mixed and not the pure quantum state, the resulting photons will not be entangled. This would mean that value of *R* depends on the material and may serve as an indicator of the intramolecular environment surrounding the positronium. In this work, we present a dedicated PET scanner, called the Jagiellonian PET (J-PET), built from plastic scintillators, in which annihilation photons interact solely via Compton scattering (with photoelectric effect at the level of 10^−5^) (*35*). In the J-PET scanner, the average distance between the primary and secondary scattering of 511-keV photon is equal to about 25 cm (compared to about 0.6 cm in crystal PET systems), rendering possible measurement of the Δφ distribution with high angular resolution of 2° (*23*). We substantiate the capability of the J-PET scanner in effectively imaging the Δφ distribution and hence for imaging of the parameter *R*, in addition to the standard PET imaging of the density distribution of annihilation points. As an example of application, we determine that the value of *R* for photons from the positron-electron annihilation in porous polymer is substantially lower than expected for maximally entangled photons, and, by comparison to the value of *R* determined for aluminum, we demonstrate that *R* is sensitive to the type of the material. This result opens up prospects for using entanglement witness *R* as a diagnostic parameter of tissue type and tissue pathology. Last, we estimate and discuss the sensitivity of the total-body J-PET scanner for the simultaneous PET and *R* value imaging in clinical diagnostics.

## RESULTS

**Fig. 2.**
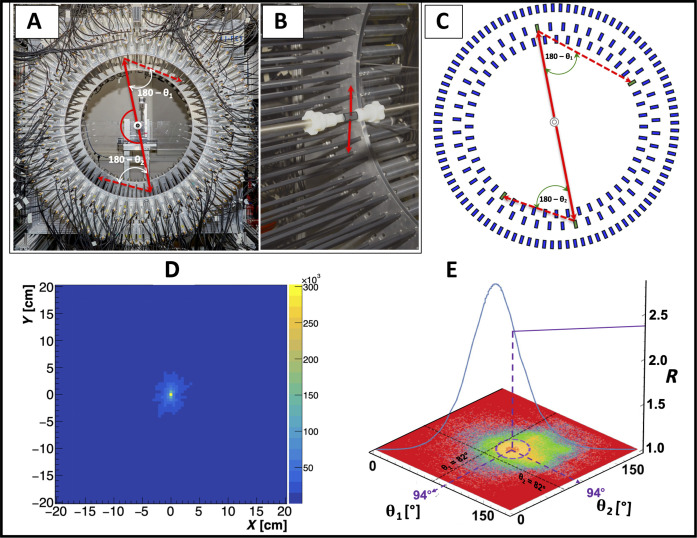
Experimental setup used in this study and exemplary distributions of registered events. (**A**) Photograph of the 192-strip J-PET tomograph with a superimposed illustration of the example event with annihilation photons (red solid arrows) and scattered photons (red dashed arrows) used in this study. (**B**) Close-up photograph of the annihilation chamber, which comprises a positron-emitting ^22^Na radionuclide surrounded by XAD-4 porous polymer (*41*). In this photograph, scintillator strips covered with black light tight foil and aluminum photomultiplier housings are visible. (**C**) The cross section of the 192-strip J-PET scanner together with the annihilation photons (red solid lines) originating from the electron-positron annihilation in the chamber and the scattered photons (red dashed lines). (**D**) Tomographic image of the annihilation source with a pixel size of 5 mm by 5 mm. (**E**) Experimentally determined distribution of scattering angles θ_2_ versus θ_1_. The maximum density of events determined at θ_1_ = θ_2_ = 94° is due to the geometry of the detector, as can be seen in (A) and (B). The superimposed blue solid curve indicates the value of entanglement witness *R* calculated for the cases where θ_1_ = θ_2_. The maximum of *R* = 2.84 is visible for θ_1_ = θ_2_ = 82°, while the experimental data concentrate around θ_1_ = θ_2_ = 94°, where *R* = 2.4.

This work experimentally demonstrates the dependence of the degree of entanglement of annihilation photons on the type of the material in which the positron annihilates and presents the capability of the newly developed J-PET scanner to image the quantum correlation of annihilation photons. Figure 2 (A and B) shows photographs of the J-PET scanner developed and constructed by the J-PET group (*35*–*38*). The technical details of the J-PET scanner and the annihilation chamber are outlined in Materials and Methods. In this section, we highlight the key features that set J-PET apart from the current PET scanners and make it capable of imaging quantum entanglement of annihilation photons. The J-PET scanner is built from plastic scintillator strips arranged axially with the photomultiplier readout at the ends. The application of plastic scintillators, instead of crystals used in the state-of-the-art PET systems, and the application of dedicated triggerless data acquisition system are the two crucial novelties enabling efficient detection of events in which both photons from *e*^+^*e*^−^ → 2γ annihilation undergo Compton scattering. In plastic scintillators, 511-keV annihilation photons interact via Compton scattering only (the fraction of photoelectric effect is at the order of 10^−5^) (*35*), and triggerless acquisition enables simultaneous detection of four interactions due to annihilation and scattered photons. Contrary to the state-of-the-art PET systems in which signal processing and acquisition are confined to two interactions only (*39*, *40*), the registration of Compton-scattered photons is mandatory for the determination of the annihilation photon’s polarization.

The linear polarization of the *i*th annihilation photon is determined using Eq. 1 (*22*, *23*). A typical topology of events used in this study is superimposed on the photograph of the scanner (Fig. 2A) and on its schematic cross section (Fig. 2C). Positron-electron annihilation occurs in the porous cross-linked styrene-divinylbenzene copolymer XAD-4 (*41*) surrounding the positron-emitting ^22^Na source placed in the annihilation chamber (Fig. 2B). The two annihilation photons are emitted in opposite directions and registered in the three-layer J-PET system, consisting of 192 plastic scintillators of 50-cm length and a cross section of 1.9 cm by 0.7 cm (*37*). The signals from photomultipliers are sampled by dedicated electronics (*42*), enabling the determination of time, position, and energy deposition for each registered photon interaction (*37*). Interactions are referred to as hits. For an event of interest, four hits are required, two hits from annihilation photons and two hits from scattered photons. Hits in the detector caused by the annihilation photons are distinguished from hits due to scattered photons based on the registered energy deposition and angular correlations. Hits from annihilation photons are used to reconstruct the tomographic image of the annihilation source. An example image, showing the reconstructed position of the source, is presented in Fig. 2D. Next, each scattered photon is assigned to the appropriate annihilation photon using information on time and positions of hits. The event selection criteria are discussed in detail in Materials and Methods. Once the hits in the event are identified, the scattering angles (θ_1_ and θ_2_) and the angle between the scattering planes (Δφ) are determined. The determined distribution of the θ_1_ versus θ_2_ angles is shown in Fig. 2E. It illustrates that the maximum of the density distribution of registered events is around the angles of θ_1_ = θ_2_ = 94°. This is due to the geometric arrangements of scintillators in the J-PET tomograph, as can be seen in Fig. 2C. Solid curve superimposed on the plot in Fig. 2E indicates the distribution of entanglement witness *R* for scatterings, where θ_1_ = θ_2_, calculated under the assumption that the annihilation photons are maximally entangled.

**Fig. 3.**
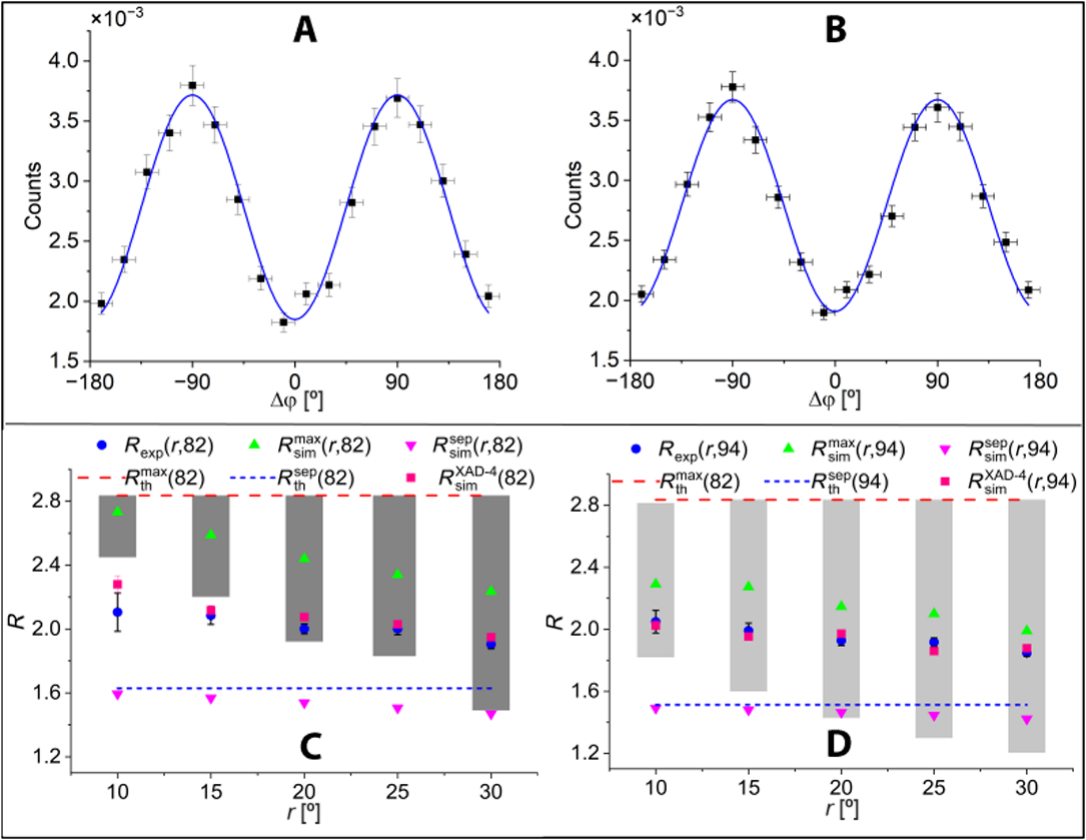
The distributions of the Δφ angle and the values of the *R* factor determined for the XAD-4 porous material. (**A** and **B**) Experimental results for events within a circle with the radius of 20° centered around θ_1_ = θ_2_ = 82° and θ_1_ = θ_2_ = 94°, respectively. The solid curve represents the best fit of the function *A*-*B*cos(2Δφ), with *A* and *B* as free parameters. Vertical bars denote statistical uncertainty, while horizontal bars indicate bin width. The tabulated data are provided in tables S1 and S2. (**C** and **D**) *R* values determined for events within circles with the radii of *r* centered around θ_1_ = θ_2_ = 82° and θ_1_ = θ_2_ = 94°, respectively (see also Fig. 4). Experimental results are shown with blue points. Gray bars denote the statistical uncertainty. The maximum possible value of *R* in the case when photons are maximally entangled (*R* = 2.84), achievable at θ_1_ = θ_2_ = 82°, is indicated by the red horizontal dashed line. The blue dashed lines indicate the values of *R* in the case when the photons are not entangled for the scatterings at θ_1_ = θ_2_ = 82° (*R* = 1.63) and θ_1_ = θ_2_ = 94° (*R* = 1.51) in (C) and (B), respectively. Upward-pointing green triangles and downward-pointing magenta triangles demonstrate *R* values determined from the simulated data. Upward-pointing green triangles correspond to the result simulated assuming maximally entangled photons, while downward-pointing magenta triangles indicate results obtained assuming that the photons are separable. The pink rectangles correspond to simulated results for porous medium XAD-4 assuming that photons from the pick-off annihilation are not entangled. The shaded boxes show the range of theoretical *R* values, in a given selected circle, calculated for the case of maximally entangled photons.

It shows that the value of *R* expected at the maximum of the density distribution of registered events is equal to *R* = 2.4, and it is lower than the maximal value of *R* = 2.84 for θ_1_ = θ_2_ ≈ 82°. For further analysis of the experimental data, we selected the region around θ_1_ = θ_2_ = 82° (112,943 events), where the highest correlation is expected, and the region around θ_1_ = θ_2_ = 94° (181,186 events), where the highest number of events is recorded. Figure 3 (A and B) shows the Δφ distribution determined for events with the θ_1_, θ_2_ scattering angles from the circle of radius *r* = 20° centered at θ_1_ = θ_2_ = 82° (Fig. 3A) and at θ_1_ = θ_2_ = 94° (Fig. 3B), respectively. The studied regions on the (θ_1_, θ_2_) plot are shown in the Materials and Methods. The formula A−Bcos(2Δφ) was fitted to the determined distributions to extract the value of $R_{\text{exp}}=\left(A+B\right)/\left(A-B\right)$. The blue curves depict the results of the fits superimposed on the data, and the resultant values of *R*_exp_ are represented by blue circles in Fig. 3 (C and D). The experimental values of *R*_exp_ that were obtained correspond to the weighted average of *R* values, where the weights are based on the density distribution of registered events over the selected region on the (θ_1_, θ_2_) distribution. In the figure, the experimental results are compared to the theoretical predictions obtained under two assumptions: (i) that the photons are maximally entangled (green triangles pointing upward) and (ii) that the photons are in a separable state (magenta triangles pointing downward). The shown theoretical predictions account for the effects of the detection system, which were modeled using Monte Carlo simulation methods, as described in detail in Materials and Methods. The measured experimental values of the entanglement witness *R*_exp_ (given in Table 1) are smaller than the predicted values for maximally entangled photons $R_{\text{sim}}^{\text{max}}$ and larger than the expected values for separable photons $R_{\text{sim}}^{\text{sep}}$. The difference between the experimental and theoretical values of the entanglement witness *R* remains consistent across all studied regions with radius *r* ranging from *r* = 10° to *r* = 30°. As the radius increases, the experimental value of *R*_exp_ decreases, and the statistical errors also decrease. For the mean radius of *r* = 20°, the determined values of *R*_exp_ for the distribution of scattering angles centered at θ_1_ = θ_2_ = 82° and θ_1_ = θ_2_ = 94° are estimated to be 2.00 ± 0.03 and 1.93 ± 0.03, respectively.

**Table 1.** Parameter *R*_exp_ determined for the range of scattering angles centered around 82° and 94° with the radius *r* = 20 cm. The values are compared to the simulations of experimental result, assuming that photons are maximally entangled ($R_{\text{sim}}^{\text{max}}$), separable ($R_{\text{sim}}^{\text{sep}}$), and originating from annihilations in XAD-4 material ($R_{\text{sim}}^{\text{XAD}-4}$).

Range radius	Range center	*R*_exp_	$R_{\text{sim}}^{\text{max}}$	$R_{\text{sim}}^{\text{sep}}$	$R_{\text{sim}}^{\text{XAD}-4}$
*r* = 20°	θ_1_ = θ_2_ = 82°	2.00 ± 0.03	2.44	1.54	2.08
*r* = 20°	θ_1_ = θ_2_ = 94°	1.93 ± 0.03	2.15	1.46	1.97

## DISCUSSION

This study demonstrates the full-scale PET scanner capable of determining the polarization of annihilation photons by registering their Compton scatterings. The scanner is constructed from three cylindrical layers of plastic scintillator strips, in which annihilation photons interact almost exclusively through Compton interaction, compared to crystals where Compton effect constitutes, e.g., 59% Bismuth Germanium Oxide (BGO) or 69% Lutetium-Yttrium Oxyorthosilicate (LYSO) (*35*, *43*, *44*). Therefore, plastic scintillators are best suited for the quantum entanglement PET system. Another crucial characteristic of the scanner presented in this work is the large relative distance between subsequent Compton interactions in plastic scintillators. In the J-PET system, it is equal, on average, to 25 cm compared to about 0.6 cm in BGO crystal scintillators. This enables one to achieve with J-PET a high angular resolution of about 2° and a high purity of up to ~95% (*23*), for the identification of first and second interactions, compared to only about 55% purity and about 6° angular resolution achieved in pixelated crystals (*34*, *45*). Moreover, in the presented J-PET system, the maximum efficiency for detecting double Compton interactions is within the angular range of 82° ± 30°, where the correlation is the highest (see Fig. 2D). Using the data collected with the J-PET scanner, we determined the distributions of the relative angle between the scattering planes of 511-keV photons (Δφ) originating from *e*^+^*e*^−^ → 2γ annihilation in the porous polymer XAD-4. The obtained shape of the Δφ distributions exhibits cos(2Δφ) oscillations with a maximum at 90°, as expected for photons with perpendicular polarizations. The experimental Δφ distributions were compared with predictions obtained under the assumptions that the photons are maximally entangled and that they are separable. The main observation reported in this work is that the correlations between the annihilation photons originating from the positron-electron annihilation in the porous polymer are larger than for the separable state, but they are smaller than expected for the maximally entangled two-photon state. To our knowledge, this finding is reported for the first time. Previous investigations of the Δφ distribution for annihilation in metals resulted in the *R* value consistent with the assumption that annihilation photons from the electron-positron annihilation are maximally entangled (*46*, *47*). The most precise experiment so far, performed for photons from positron-electron annihilation in aluminum, yielded *R* = 2.435 ± 0.018 (*24*), which, taking into account the detector geometrical acceptance, is consistent with expectations for the maximally entangled two-photon state (*24*). In metals, positrons exclusively annihilate directly with electrons (*9*), whereas in the porous polymer XAD-4 used in this study, positrons annihilate directly in only 32% of cases, and in the remaining 68% of cases, the annihilation proceeds through the formation of positronium atoms (*41*). In this study, the conversion of oPs on oxygen molecules is suppressed because air has been pumped out of the XAD-4 material to 10^−4^ Pa using the dedicated chamber and the vacuum system (*48*). We hypothesize that the nonmaximal entanglement can be attributed to the annihilation of positrons with electrons bound to the molecules, when a positron from positronium annihilates with an electron from the surrounding atoms (*17*–*19*). In general, when annihilation is from the mixed state, the entanglement may be partially or fully lost (*20*, *49*). Using Eq. 7 and taking into account that, in the case of XAD-4 material, the pick-off process constitutes 31% of 2γ annihilations (see Table 2), we obtain *R*(XAD-4) = 2.36. Pink squares in Fig. 3 (C and D) show the prediction for the measured value *R* simulated using the value of *R*(XAD-4) = 2.36, taking into account the properties of the detection system and the data selection criteria. The result obtained under the assumption that photons from the pick-off process are not entangled (pink squares) is in quite good agreement with the experimentally determined values of *R* (blue circles). In Table 1, the calculated and measured values are compared for *r* = 20°. To further explore the origin of the observed nonmaximal entanglement, dedicated experiments are required in which the annihilation mechanism can be identified, e.g., by additional measurements of the positron lifetime. The observation reported in this work, based on measurements in the XAD-4 porous polymer, demonstrates that the degree of entanglement of annihilation photons, expressed via the *R* parameter, is not maximal. This observation can be explained by assuming that photons from the pick-off annihilations are not entangled. The rate of the pick-off process in matter is determined by the size of the pores (free voids between atoms) in which positronium atoms are formed. The smaller the size of the free voids, the larger the pick-off contribution. It is well established that the larger the pick-off rate, the smaller the positronium lifetime (*9*). Here, we anticipate that the *R* value will also be smaller. The oPs lifetime is known to vary with tissue type, and it has been argued that the observed lifetime changes are predominantly due to the differences in the molecular tissue structure (*9*). Therefore, the result of this work opens perspectives to apply *R* as a new diagnostic indicator that may be available in PET imaging. The nonmaximal entanglement of annihilation photons observed in this work, created in electron-positron annihilation in porous polymer, is well explained by the assumption that photons from the pick-off process are not entangled, while photons originating from direct and pPs annihilations are maximally entangled. However, the assumption that photons from direct annihilation are maximally entangled is based on previous measurements of the *R* value in aluminum (*24*), and the maximal entanglement of photons from pPs annihilation is predicted theoretically (*14*) but not yet confirmed experimentally. Thus, the main limitations of the studies presented in this article are the lack of *R* value measurements for different materials with the same detector system and the lack of disentanglement between different annihilation mechanisms. Therefore, further systematic measurements with different materials are required to understand how variations in molecular structure affect the *R* value. The parameter *R* has been used as an entanglement witness in annihilation photon studies to date. Recently, a strategy based on the fact that the analyzing powers and measure of correlations in the visibility function can be factorized was put forward in (*50*), which allows for the extraction of these dependencies and thus the definition of a measure of entanglement degree independent of the scattering angle. The method proposed in (*50*) could reduce the required statistics and facilitate the comparison of results from different experiments. In the future, we intend to apply this approach to the J-PET detector. Moreover, to unambiguously answer the question of how the Δφ distribution (and hence the *R* value) depends on the annihilation mechanism, the registration of prompt gamma, in addition to annihilation photons, will be required. Simultaneous registration of annihilation photons and prompt gamma will enable the determination of the positron lifetime in the studied material and hence will enable to disentangle annihilations proceeding directly, via pPs or via pick-off processes.

**Table 2.** The fraction of the main processes leading to annihilation of positrons in XAD-4 materials.

Process	Direct (*e*^+^*e*^−^ → 2γ)	pPs → 2γ	oPs → 2γ (pick-off)	oPs → 3γ
**Total fraction (%)**	32	17	22	29
**Fraction of** 2γ **(%)**	45	24	31	0

The imaging of polarization correlations requires coincident registration of four interactions and therefore needs high-sensitivity scanners capable of multiphoton registration. New-generation PET scanners covering the whole body provide high enough sensitivity (*51*–*53*), yet for imaging of polarization correlation, a multiphoton acquisition is required, which is not yet available in the current clinical PET systems. In the case of crystal PET systems, multipixel readout will also be necessary (*32*, *34*, *45*). The first total-body PET scanner based on plastic scintillator is under construction at Jagiellonian University using J-PET technology (*35*). With the extended 250-cm field of view, this scanner will offer high multiphoton registration efficiency, enabling not only conventional 2γ metabolic PET imaging with high statistical accuracy but also polarization correlation imaging and using the recently developed positronium imaging (*54*, *55*) as a new diagnostic biomarker.

## MATERIALS AND METHODS

### Positron annihilation chamber

In the reported experiment, positrons were annihilating in the XAD-4 (CAS 37380-42-0) material (*41*). Amberlite XAD-4 is a porous polymer resin with a high surface area and a porous structure, in which large fraction (68%) of positron annihilations proceeds via positronium formation (*41*). In the XAD-4 polymer, positron annihilation proceeds in approximately 32% via the direct *e*^+^*e*^−^ → 2γ process, in approximately 17% via *e*^+^*e*^−^ → pPs → 2γ, in approximately 29% via *e*^+^*e*^−^ → oPs → 3γ, and in 22% via *e*^+^*e*^−^ → oPs, followed by oPs pick-off annihilation into 2γ. Thus, the two-photon annihilations proceed in 45% via direct *e*^+^*e*^−^ annihilation, in about 24% via self-annihilation of pPs, and in about 31% via the pick-off process of oPs (Table 2). The ^22^Na isotope with an activity of 1.1 MBq was used as a positron emitter. The ^22^Na source was sandwiched between two 7-μm–thick kapton foils and enclosed on both sides by a few-millimeter-thick layer of the XAD-4 porous polymer. The polymer and the positron source were situated within a dedicated chamber connected to a vacuum system that enabled the evacuation of air from the polymer pores to a pressure of 10^−4^ Pa (*48*). The chamber was placed at the center of the J-PET scanner as illustrated in Fig. 2B. The chamber was constructed from 1-mm–thick polyamide 6 with a density of 1.14 g/cm^3^, resulting in a negligible absorption (less than 1%) of 511-keV photons (*48*). It is worth noting that a high vacuum was essential to suppress mechanisms that can be triggered by the presence of paramagnetic oxygen molecules, such as the conversion of oPs to pPs, which disrupts the oPs decay dynamics by decreasing the oPs lifetime and also by decreasing the fraction of pick-off processes. However, vacuum is not required for quantum entanglement studies, and therefore, a similar methodology could be adapted for in vivo studies where differences in molecular composition can affect entanglement properties through different ratios of pick-off annihilations to direct and pPs annihilations.

### J-PET scanner

J-PET is the first PET tomograph composed of plastic scintillators in which the Compton interaction is the main photon registration process (*36*). The scanner used in this work consists of 192 plastic scintillators (EJ-230; 50 cm by 1.9 cm by 0.7 cm) forming three concentric cylindrical layers (see Fig. 2A) (*37*). The scintillators are connected at the ends to the vacuum tube photomultipliers (Hamamatsu, R9800). The time and position of the interaction of photons (referred to as hits) along the scintillators are estimated by measuring the time of light signal arrivals to the photomultipliers (*36*). The signals from the photomultipliers are sampled at four fixed thresholds (30, 80, 190, and 300 mV) using time-to-digital converters implemented in field-programmable gate array devices (*42*). The time stamps of the signals were recorded in the triggerless mode using the dedicated data acquisition system, which can process data streams at a speed of about 8 Gbps (*56*). The calibration procedure and intrasynchronization of the timing signals between 192 detection modules are explained in previous work. The hit time and hit position resolutions are equal to 250 ps and 25 mm, respectively (*37*). The maximum energy deposited by a 511 keV inside a plastic scintillator corresponds to 340 keV, with an energy resolution of about 7.5%. The angular resolution for determining the scattering angle of annihilation photon amounts to 2° (*23*).

### Event selection and classification

**Fig. 4.**
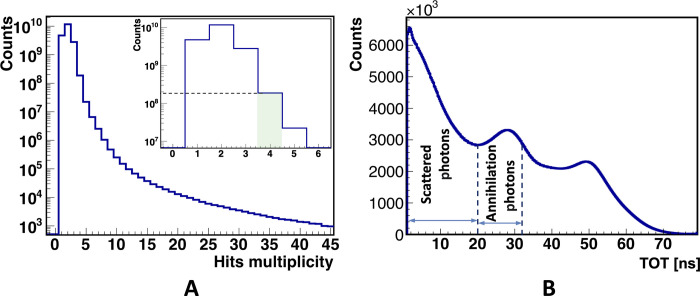
Experimental distributions used in the data selection. (**A**) Distribution of hit multiplicity in events. Inset highlights four-hit events used for the analysis in this work. The number of events with a multiplicity of 1 is suppressed by prescaling the data with single hits in the analysis. (**B**) TOT histogram for all hits. Slopes at TOT values of about 32 and 56 ns correspond to the Compton edges, resulting in maximum energy deposition by 511-keV annihilation photons and 1275-keV prompt gamma (from ^22^Na decay), respectively. Vertical dashed lines indicate the range of TOT values used to select candidates for annihilation photons (20 to 32 ns) and scattered photons (1 to 20 ns).

The data were collected continuously for 122 days and analyzed using a dedicated framework developed based on C++ and Root (a data analysis tool developed at CERN) (*57*) with detector-specific and advanced features. Events useful for the study of the polarization correlations comprise four hits within 20-ns time window, two hits caused by primary photons (511 keV) and the remaining two hits caused by the corresponding scattered photons with lower energy (e.g., 275 keV for the scattering at 82°). An example of these events is shown in Fig. 2 (A and C). Figure 4A shows the histogram of the hit multiplicity in the event. For the analysis, only events with multiplicity equal to 4 were accepted (total events = 1,070,127,000). In the next step of analysis, as a first criterion to disentangle between the annihilation and scattered photons, the time-over-threshold (TOT) value was used, which is a measure of the energy deposition (*38*). For the 511-keV annihilation photons, the energy depositions varies between 0 and 341 keV, and for the scattered photons of interest, e.g., for the photons scattered at angles in the range of 82° ± 30°, the maximum energy deposition is equal to 218 and 98 keV for photons scattered under 52° and 112°, respectively. Figure 4B shows the distribution of TOT with superimposed vertical lines, indicating ranges chosen for selecting scattered photons candidates (1 ns < TOT < 20 ns) and annihilation photon candidates (20 ns < TOT < 32 ns). The values of TOT are uniquely correlated with the energy deposition and, for example, TOT = 32 ns corresponds to 341 keV (Compton edge for 511-keV photons), and TOT = 56 ns corresponds to 1062 keV (Compton edge for 1275-keV gamma from ^22^Na decay) (*58*). Furthermore, for the annihilation photon candidates, the stringent back-to-back (175° < θ < 185°) emission criterion was applied.

**Fig. 5.**
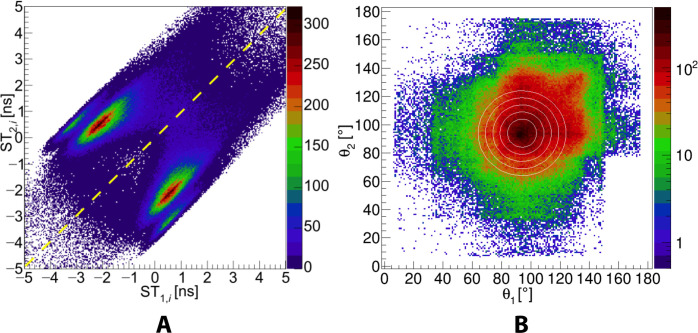
Experimental distributions used for events classification. (**A**) Distribution of ST_2*i*_ versus ST_1*i*_ used to assign the *i*th scattered photon to the first or second annihilation photon. Yellow diagonal dashed line is used as a criterion for the selection. If the (ST_1*i*_,ST_2*i*_) point is below the diagonal, then it is assumed that the *i*th photon is a result of the scattering of the first annihilation photon. Contrary, if the (ST_1*i*_,ST_2*i*_) point is above the diagonal, then the *i*th scattered photon is assigned to the second annihilation photon. The observed split structure is due to the arrangement of the scintillators (Fig. 2). (**B**) Experimental distribution of the θ_2_ versus θ_1_ angles. Because of the geometrical arrangement of scintillators in the J-PET scanner (Fig. 2), the maximum density of the (θ_2_,θ_1_) distribution is for the scatterings around the angles of θ_1_ = θ_2_ = 94°. The white circles indicate regions with the radii of *r* = 10°, 15°, 20°, 25°, and 30° used in the analysis (see Fig. 2).

Moreover, to ensure that photons are emitted from the source, an information from the tomographic image (Fig. 2D) was used. The image shows the density of annihilation points reconstructed on the basis of the times and positions of hits identified as originating from annihilation photons. For further analysis, only events were considered for which the distance between the reconstructed annihilation site and the center of the image (position of the source) was less than 1 cm in the *xy* plane and less than 4 cm in the *z* direction. After selecting the hits in the event corresponding to the annihilation photons, as a next step, the hits due to the scattered photons are assigned to the proper annihilation photon. By assigning indices 1 and 2 to the hits from annihilation photons and indices 3 and 4 to the hits from scattered photons, the next step in the analysis can be defined as testing two hypotheses: (i) The third photon is a result of scattering from the first annihilation photon and the fourth photon is a result of scattering from the second annihilation photon, and (ii) the third photon is a result of scattering from the second annihilation photon and the fourth photon is a result of scattering from the first annihilation photon. To test the hypothesis stating that *i*th photon is a scatter of the *j*th photon, we define a “Scatter Test” value: ST_*j*,*i*_ = Δ*t*_*j*,*i*_ − *r*_*j*,*i*_/*c*, where $\Delta t_{j,i}=t_{j}^{\text{annihilation}}-t_{i}^{\text{scatter}}$ and *r*_*i*,*j*_ is the distance between the hit positions of the annihilation photon (*r*_*j*_) and scatter photon (*r*_*i*_). ST is a difference between the measured time of flight of the scattered photon and the time of flight calculated for the light to travel between the *i*th and *j*th hits. In the case of ideal time and position resolution of the scanner, if the *i*th photon is a scatter of the *j*th photon, then ST must be equal to zero. Figure 5A shows the distribution of values ST_1,*i*_ versus ST_2,*i*_ for testing the hypotheses whether the *i*th scatter photon originates from first or second annihilation photon. If the point (ST_2,*i*_,ST_1,*i*_) on the plot is below the diagonal, then the *i*th scatter photon is assigned to the first annihilation photon. If it is above the diagonal, then it is assigned to the second annihilation photon. For the final analysis, only these events were selected for which each of the two different scattered photons was assigned to a different annihilation photon.

After applying the sequential selection criteria to isolate events of interest, the number of remaining events was reduced (relative to the total number of initial events) to 92% following the axial restriction on the active length of scintillators, 19% after selecting annihilation and scattered photons based on the TOT, 2% for selecting events containing exactly two annihilation photons and two scattered photons, 0.1% after reconstructing the annihilation coordinates and ensuring that annihilation events originated from the source chamber, and 0.05% after applying scattering test to correctly pair each scattered photon with its corresponding primary photon. Last, for each event, the values of scattering angles θ_1_ and θ_2_ and the relative angle between the scattering planes (Δφ) are determined. Figure 5B shows the distribution of scattering angle θ_2_ versus θ_1_. The maximum density of registered events is observed for θ_2_ = θ_1_ = 94°. This is due to the geometrical arrangement of scintillators in the J-PET scanner, as can be seen in Fig. 2 (A and C). The white circles superimposed on the experimental distribution in Fig. 5B illustrate angular regions centered at θ_2_ = θ_1_ = 94°, for which the Δφ distributions and *R* parameter were determined and presented in Fig. 3D.

### Estimation of J-PET scanner acceptance and registration efficiency

The correction of the measured distributions for the geometrical acceptance and registration efficiency of the J-PET scanner is one of the most crucial steps in determining the polarization correlation of annihilation photons. Efficiency and acceptance were simultaneously determined in this work, and, for the sake of brevity, we will refer to this combined quantity as “efficiency” throughout the manuscript. Thus, in the following, we will understand by efficiency the combined geometric acceptance, detection efficiency, and event selection efficiency. This defined efficiency was estimated as a function of the Δφ angle using the GEANT4 (GEometry ANd Tracking) simulation package (*59*). For this purpose, the full scanner geometry and material composition (*37*) were defined in the GEANT4 structure, and the *e*^+^*e*^−^ annihilations were simulated in the center of the detector, assuming the fraction of direct annihilations, pPs and oPs formations, as it is known for the XAD-4 porous polymer (*41*). The method of simulations with the J-PET scanner was validated in the previous works, as described, e.g., in (*23*, *60*). The response of the scanner was simulated. Next, the simulated data were analyzed using the same criteria as applied to the experimental data. Then, these determined Δφ distributions were normalized to the original Δφ distributions determined by simulating the annihilation photon interactions in the scanner and taking for the calculations of true scattering angles (*23*). The resulting normalized Δφ spectra were used to correct the experimental Δφ spectra for efficiency. Example of corrected Δφ spectra is shown in Fig. 3 (C and D).

### Simulations of Δφ distributions for separable and entangled annihilation photons

For simulations performed in this work, it was assumed that the polarizations of the back-to-back propagating annihilation photons are perpendicular to each other. For the separable state, the interaction of each photon was simulated independently using the GEANT4 package, in which the Compton scattering is simulated according to the Klein-Nishina formula (*21*). To simulate the distribution for entangled photons, we used the data simulated for separable state and preselected events such that the resulting distributions are as expected for the entangled photons. The method used for simulations of separable and quantum entangled photons was described in detail and validated in (*23*).

### Systematic uncertainties

The precision of the *R* value achieved in this study, at the level of 10^−2^, is primarily limited by statistical uncertainties, while the systematics of the detector performance, as already demonstrated in previous works, is well controlled up to the level of at least 10^−4^ (*23*, *35*, *38*, *60*, *61*). The measurement was purposely performed with a very low activity source of 1.1 MBq, resulting in accidental coincidences of less than 2%. However, this was achieved at the cost of a very long measurement time of 122 days. A signal event is composed of four hits registered in four different scintillator strips (2γ + 2γ′). The most important feature of the detector is that, in a given layer, each scintillator contributes equally to the registration of all configurations. Therefore, even if the efficiency of a given scintillator was not well estimated, only the statistics would be decreased; the shape of the Δφ distribution, however, would not be changed. In addition, only the shape is important for the results of these studies. In total, 192 scintillator strips with an angular distance of 1.875° are located at an average radius of ~49.5 cm. In a given layer, each single strip contributes equally to the final result. For the sake of argument, the full removal of a single detector (1 of 192) would have a 5 × 10^−3^ effect on the statistics, whereas an exaggerated misplacement of a strip by 0.1 cm would have a 0.14° effect, while the angular coverage of a single strip in the *xy* plane is 0.5°. So, even if the efficiency and geometry of a given scintillator were determined with a precision of 10^−3^, the effect on the total efficiency would be at the level of 10^−5^. However, the total efficiency does not influence the accuracy of the *R* determination. Here, the crucial factor is the relative efficiency between the registration of various Δφ angles. In addition, because each scintillator contributes to the measurement of all configurations, the *R* determination is not affected by inaccuracies in the efficiency determination of single detector strips. In addition, with the possibility of image reconstruction, the average position of the source can be controlled within 0.5 mm in the *xy* plane and 0.4 mm along the *z* axis, while the thickness of the scintillators is 7 mm. The influence on the Δφ distribution and *R* extraction of the effects of primary and secondary photon identification, assignment of secondary to primary photons, and, in general, the influence of all criteria used in the data selection process were tested by changing these criteria and reanalyzing the data. In all cases, no statistically significant change in the result was found. It is worth mentioning that the full geometry of the J-PET detector, including all the aluminum elements of the mechanical construction for possible scattered events, was simulated. No influence of these events was found. Figure 3 (C and D) shows that the averaging of *R* over the angles, simulated and measured, follows the same pattern and that the conclusion of this article does not change when the range of θ_1_ versus θ_2_ angles is changed by using a different range of scattering angles.
