# Supplementary material for: Nonmaximal entanglement of photons from positron-electron annihilation demonstrated using a plastic PET scanner
Source: Sci Adv. 2025 Apr 30;11(18):eads3046. doi: 10.1126/sciadv.ads3046 (PMC12042903; doi:10.1126/sciadv.ads3046)
Supplement: Supplementary file 1 — Tables S1 and S2 [file sciadv.ads3046_sm.pdf]

Science Advances
AAAS

Supplementary Materials for

Nonmaximal entanglement of photons from positron-electron annihilation demonstrated using a plastic PET scanner

Paweł Moskal *et al.*

Corresponding author: Paweł Moskal, p.moskal@uj.edu.pl; Sushil Sharma, sushil.sharma@uj.edu.pl

Sci. Adv. **11**, eads3046 (2025)
DOI: 10.1126/sciadv.ads3046

This PDF file includes:

Tables S1 and S2

x	y	x_{error}	y_{error}
-170	0.001980	10	8.80731E-5
-150	0.002350	10	1.08522E-4
-130	0.003070	10	1.40798E-4
-110	0.003400	10	1.50043E-4
-90	0.003800	10	1.65898E-4
-70	0.003470	10	1.5127E-4
-50	0.002850	10	1.25534E-4
-30	0.002190	10	9.62704E-5
-10	0.001830	10	7.99024E-5
10	0.002060	10	9.06109E-5
30	0.002130	10	9.34263E-5
50	0.002820	10	1.22733E-4
70	0.003450	10	1.51747E-4
90	0.003690	10	1.60406E-4
110	0.003470	10	1.53699E-4
130	0.003000	10	1.37354E-4
150	0.002390	10	1.07997E-4
170	0.002040	10	9.24511E-5

Table S1: Data for $\Delta\varphi$ distribution for scattering angles centered at 82° with a radius of 20° shown in Fig.3(a)

x	y	x_{error}	y_{error}
-170	0.002050	10	6.73727E-05
-150	0.002340	10	7.72558E-05
-130	0.002970	10	9.95061E-05
-110	0.003520	10	1.20182E-04
-90	0.003780	10	1.26624E-04
-70	0.003340	10	1.10531E-04
-50	0.002860	10	9.09897E-05
-30	0.002320	10	7.32264E-05
-10	0.001900	10	5.76884E-05
10	0.002090	10	6.47560E-05
30	0.002220	10	6.93346E-05
50	0.002700	10	8.63291E-05
70	0.003440	10	1.13132E-04
90	0.003610	10	1.20163E-04
110	0.003450	10	1.15259E-04
130	0.002870	10	9.61277E-05
150	0.002480	10	8.24528E-05
170	0.002090	10	6.88927E-05

Table S2: Data for $\Delta\varphi$ distribution for scattering angles centered at 94° with a radius of 20° shown in Fig.3(b)
